# Comment on “Variability in the Branching Pattern of the Internal Iliac Artery in Indian Population and Its Clinical Importance”

**DOI:** 10.1155/2016/8193713

**Published:** 2016-12-26

**Authors:** Vinodhini Bhaskaran

**Affiliations:** Sri Kota Specialist Medical Centre, Jalan Mohet, 41000 Klang, Selangor Darul Ehsan, Malaysia

I recently came across an interesting and useful article on “Variability in the Branching Pattern of the Internal Iliac Artery in Indian Population and Its Clinical Importance,” by Sakthivelavan et al. [1]. As a practicing obstetrician who has served in several parts of developing countries, I would first like to thank the authors for their commendable desire to contribute to a topic of great clinical significance. Hemorrhage is one of the common causes of maternal deaths in the developing nations [2, 3]. Pelvic hemorrhage is also a major cause of morbidity and mortality in gynecological surgeries [4]. Kelly [5] was the first to describe ligation of the internal iliac artery (IIA) as a method to control hemorrhage during pelvic surgery in 1894. Since then, this procedure has helped save many lives and uteruses for over a century.

It was long believed that ligation of the hypogastric arteries would lead to complete cessation of blood flow in the area supplied by these vessels. Yet, owing to the activation of the anastomotic network immediately after ligation, blood is never completely drained from the hypogastric artery distally to the site of ligation as demonstrated by Burchell in 1968 [6]. Bleeding from the uterus diminishes because there is no arterial pressure or pulsation in the arteries after ligation; instead, pressure becomes the same as that in the venous system.

The use of IIA ligation in current obstetrics and gynecology is controversial due to the technical challenges the procedure involves and the variation in success rates. The efficacy of this procedure in controlling obstetrical hemorrhage has been reported to range within 42–75% [7–10]. Despite the fact that this procedure is mostly done as an emergency, it still requires the surgeon to have a thorough understanding of the anatomy to prevent iatrogenic injury and to have adequate hemostasis. It was interesting to read about the different anatomical variations of the IIA in the Indian population. Conventionally, the IIA is ligated 5 cm distal to the bifurcation of the common iliac artery as it is said to spare the posterior division and avoid gluteal ischemia and necrosis. However, I was quite surprised to see that in as high as 25.9% of the cases this might not be the case due to anatomical variation [1]. It was also interesting to note the anomalous origin of the obturator artery from the posterior division in 6.8% of cases [1], as the parietal branches of the obturator play an important role in collateral circulation. Knowledge about these anatomical variations is of paramount importance to the operating surgeons, as it would help prevent further blood loss and other potentially severe complications such as ureteral injury, vein laceration, and ligation of the external iliac artery. This also explains the variation in the success and complications rates in different studies.

Though the IIA ligation is less performed these days, it still continues to be a surgical alternative in cases of pelvic hemorrhage and any surgeon who encounters this needs to be aware of the anatomy of the IIA and its regional variations so that the desired hemostasis is achieved with minimal complications.
